# Field-Effect Transistors Based on Single-Layer Graphene and Graphene-Derived Materials

**DOI:** 10.3390/mi14061096

**Published:** 2023-05-23

**Authors:** Octavian-Gabriel Simionescu, Andrei Avram, Bianca Adiaconiţă, Petruţa Preda, Cătălin Pârvulescu, Florin Năstase, Eugen Chiriac, Marioara Avram

**Affiliations:** 1 National Institute for Research and Development in Microtechnologies—IMT Bucharest, 126A Erou Iancu Nicolae, 077190 Voluntari, Romania; 2 Faculty of Applied Chemistry and Material Science, University Politehnica of Bucharest, 313 Splaiul Independenţei, 060042 Bucharest, Romania

**Keywords:** field-effect transistor (FET), single-layer graphene (SLG), graphene/graphite nanowalls (GNW), bulk nanocrystalline graphite (bulk-NCG)

## Abstract

The progress of advanced materials has invoked great interest in promising novel biosensing applications. Field-effect transistors (FETs) are excellent options for biosensing devices due to the variability of the utilized materials and the self-amplifying role of electrical signals. The focus on nanoelectronics and high-performance biosensors has also generated an increasing demand for easy fabrication methods, as well as for economical and revolutionary materials. One of the innovative materials used in biosensing applications is graphene, on account of its remarkable properties, such as high thermal and electrical conductivity, potent mechanical properties, and high surface area to immobilize the receptors in biosensors. Besides graphene, other competing graphene-derived materials (GDMs) have emerged in this field, with comparable properties and improved cost-efficiency and ease of fabrication. In this paper, a comparative experimental study is presented for the first time, for FETs having a channel fabricated from three different graphenic materials: single-layer graphene (SLG), graphene/graphite nanowalls (GNW), and bulk nanocrystalline graphite (bulk-NCG). The devices are investigated by scanning electron microscopy (SEM), Raman spectroscopy, and *I*-*V* measurements. An increased electrical conductance is observed for the bulk-NCG-based FET, despite its higher defect density, the channel displaying a transconductance of up to ≊$4.9\times 10^{-3}$ A V${}^{-1}$, and a charge carrier mobility of ≊$2.86\times 10^{-4}$ cm${}^{2}$ V${}^{-1}$ s${}^{-1}$, at a source-drain potential of 3 V. An improvement in sensitivity due to Au nanoparticle functionalization is also acknowledged, with an increase of the ON/OFF current ratio of over four times, from ≊178.95 to ≊746.43, for the bulk-NCG FETs.

## 1. Introduction

Field-effect transistors (FETs) have attracted extensive attention in biosensing applications. Unlike traditional sensing methods, FET-based biosensors do not involve extensive photodetectors or fluorescent labeling parameters. A biosensor has two key components: a bioreceptor and a transducer. The bioreceptor is a biomolecule that generates a physical or chemical reaction upon interaction with a target analyte in a biological media. The transducer primarily converts the identification process into a measurable signal, which can be either electrical, electrochemical, optical, or mechanical. One electric-type biosensor that has gained a significant amount of traction over the last few years is the FET. The FET biosensor is composed of a substrate with source, drain, and gate electrodes, as well as a conductive channel that is functionalized with a bioreceptor (e.g., linker molecule) and acts as the transducer. The bioreceptor–analyte reactions will cause a change in the electrical conductivity of the transducer, and the FET’s biosensitivity occurs when the respective change in conductivity is large enough to be measured [1,2,3].

As the size and performance of silicon transistors have approached their physical limits, it is necessary to look for alternative materials to support emerging technologies. One of these materials is graphene, which is an attractive option for the source-drain channel of field effect transistors due to its excellent electrical, optical, mechanical, and thermal properties [4]. In recent years, field-effect transistors based on graphene and graphene-derived materials (GFETs) have received an increasing amount of attention owing to their unique properties, such as high sensitivity and precision, low cost, ease of surface functionalization, low operating power requirement, and miniaturization [5]. The GFETs are an improved alternative to conventional metal-oxide-semiconductor field effect transistors (MOSFETs) due to their high surface-to-volume ratio, unique electrical properties, high sensitivity, good chemical stability, and biocompatibility, making them suitable for biomolecule detection [6].

As with traditional silicon-based FETs, the GFET’s gate modulates the flow of electrons or holes in the channel. Due to the nanoscale thickness of the GFET’s channel, the current flows mainly on its surface; therefore, the GFETs possess a high sensitivity. The higher electrical and thermal conductivity of graphene results in reduced losses and enhanced heat dissipation compared to silicon. As a result, graphene-based transistors have the potential to deliver enhanced performance and efficiency [7,8,9].

In the literature, the term *graphene* is used as a general definition for graphene materials, such as graphene (single or multilayer), graphene oxide, or reduced graphene oxide [10,11]. However, the definition of the specific material is an important step for establishing the device’s properties and the material properties’ influence over device functionality. Graphene, an allotrope of carbon, consists of a single layer of atoms organized in a two-dimensional honeycomb lattice structure [12]. Single-layer graphene (SLG) was investigated theoretically by P. R. Wallace in 1947 [13] and was first produced and recognized in 2004 by Geim and Novoselov [14]. Alternative carbonic materials with graphene as a common base building block, such as nanocrystalline graphite/graphene (NCG), can also be used in sensor integration for ease of production. Bulk-NCG and graphene/graphite nanowalls (GNW) thin films are both graphenic materials and part of the wider NCG class, thus retaining much of graphene’s properties [15]. Their internal structure consists of sp${}^{2}$ crystalline nanodomains and is similar for both of the carbonic films. Their intrinsic structure similarities and association with the NCG class can be ascertained from their comparable Raman spectra. The distinguishing features between them are the surface morphology and the general orientation of the graphitic c-axis with respect to the substrate. While bulk-NCG films contain turbostratic nanocrystalline domains with the c-axis orientation tending to a perpendicular direction to the substrate, GNW films consist of similar nanodomains but with the c-axis favoring an orientation closer to a parallel direction with respect to the substrate. The nanodomains in bulk-NCG are also more densely packed, resulting in a smoother, low-roughness surface. On the other hand, GNW films are arranged as perpendicular graphitic planes on the substrate with a high specific surface area, depending on deposition conditions [15,16]. Over the years they have been known by several denominations, such as carbon nanowalls (CNW), vertical graphene (VG), GNW, and others [17].

One of the first devices to employ graphene-derived materials for biosensing applications was reported by Mohanty and Berry in 2008. They produced cost-efficient chemically modified graphene-based transistor-like devices for the purpose of bacterial and DNA detection with enhanced sensitivity tunability [18]. Exosome detection was also reported for an SLG-based FET, where the authors observed a shift in the Dirac point of the *I*-*V* curve with respect to the gate voltage [19].

Gold’s standard potential of 1.52 V makes Au nanoparticles (AuNPs) more stable from an electrical standpoint compared to other noble metal nanoparticles such as AgNPs (Ag having a standard potential of 0.79 V) [20]. Their high stability makes AuNPs a prime candidate for sensor functionalization in biodetection applications. Additionally, their optical properties, together with excellent biocompatibility, potentiated electron transfer, increased plasmonic activity, and ease of functionalization, make them promising for biomedical applications [21].

The feasibility of terahertz operation was also determined in 2012 by Mehr et al., with a transistor based on vertically orientated graphene sheets [22]. Later, in 2013, both the exceptional sensitivity as well as the enhanced selectivity of an FET biosensing device based on such a graphenic material decorated with AuNPs was demonstrated by Mao et al. [23]. A more recent study, reported in October 2020, shows the detection of DNA and streptavidin ($10^{-18}$ mol L${}^{-1}$) using an FET based on graphene decorated with AuNPs [8].

**Figure 1:**
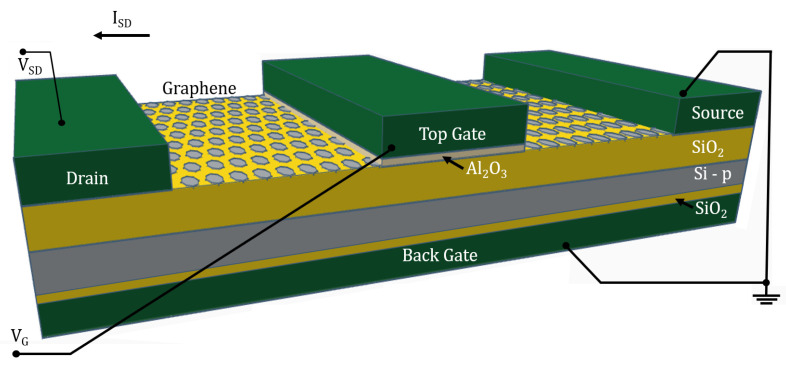
The structure and electrical scheme of the double gate graphenic field-effect transistor (GFET).

The GFETs reported in this paper are four-terminal devices (Figure 1), similar to conventional FETs in some aspects. They consist of a source electrode, a drain electrode, a top gate, and a back gate. We present here for the first time, a comparative experimental study for FETs with channels fabricated from three different graphene-based materials: SLG, GNW, and bulk-NCG. Some of the GFETs are decorated with AuNPs to take advantage of benefits such as an increased surface-to-volume ratio and an improved detection limit. As the fabricated GFETs are intended for biosensing applications, the graphenic channel is left exposed for both the initial AuNPs functionalization as well as for an ulterior anti-body conjugation of said NPs. Moreover, the graphenic channel will require direct contact with an analyte solution in order for the GFET to fulfill its intended purpose of a biosensing device.

## 2. Properties of the Graphenic Materials

The greater part of their most noteworthy features resides in their electrical properties. Generally, graphene has a very low resistivity at room temperature, as well as high intrinsic mobility, over 100 times that of Si. Theoretically, graphene can transfer electric current with 100% efficiency, and even the earliest manufactured graphene layers, although prepared using relatively modest methods in limited controlled conditions, presented remarkable qualities with reasonably high quasiparticle mobility for FET-like devices (up to 5000 cm${}^{2}$ V${}^{-1}$ s${}^{-1}$) [24]. Over the years, the transfer process has improved considerably [25], and thus mobilities of over 10${}^{4}$ cm${}^{2}$ V${}^{-1}$ s${}^{-1}$ were achieved on various substrates (e.g., SiO${}_{2}$, h-BN) [26] and over 10${}^{5}$ cm${}^{2}$ V${}^{-1}$ s${}^{-1}$ for suspended graphene samples [27,28]. For films directly grown on a substrate, such as the GNW and bulk-NCG thin films, the mobility is reduced due to dispersion caused by surface roughness, blocked load, defects, and others [26]. Their distinct molecular structure and morphology also prove advantageous for electrochemical and biosensors. On top of their excellent electrical properties, graphenic materials also possess high thermal conductivity. The thermal conductivity of SLG at room temperature can reach values of 600 W m${}^{-1}$ K${}^{-1}$ on a SiO${}_{2}$ substrate [29] and up to 5300 W m${}^{-1}$ K${}^{-1}$ for suspended SLG [30]. As it is extremely thin, the reflectivity of a graphene layer is <0.1% in the visible region and increases to ≊2% for 10-layer graphene [31]. Despite its atomic thickness, graphene is still visible and can absorb approximately 2.3% of white light, and its opacity increasing with the number of layers [31,32]. Graphene is also an inert material that does not react with other materials readily. However, under certain conditions, it can adsorb different molecules and atoms that alter its properties. Such characteristics and material performances make graphene and graphenic materials an ideal candidate for use in field effect transistors [25,33].

## 3. Materials and Methods

### 3.1. Processes for Growing the Three Types of Graphenic Materials

The SLG thin films were grown on Cu substrates by Chemical Vapor Deposition (CVD), while the GNW and bulk-NCG films were grown directly on SiO${}_{2}$ substrates through plasma-enhanced CVD (PECVD). All graphenic films presented here were grown with the Nanofab1000 equipment from Oxford Instruments, UK, and the specific process parameters are presented in Table 1.

The SLG thin films are grown via thermal CVD on a catalyst substrate, which consists of 35 μm thick Cu foils of 99.95% purity (Graphene Platform Corporation, Tokyo, Japan), with methane (CH${}_{4}$) and hydrogen (H${}_{2}$) as precursors. Following the CVD process, the films are transferred onto SiO${}_{2}$ substrates for manufacturing the GFET’s channel by wet chemical processes [34,35,36].

The GNW and bulk-NCG films used for the GFETs’ fabrication are grown via PECVD in a capacitively coupled RF reactor from CH${}_{4}$, with the addition of H${}_{2}$ or argon (Ar), respectively. The deposition technique offers the advantage of growing the films directly on an insulating layer (i.e., SiO${}_{2}$), thus eliminating the need for an ulterior transfer step, which, for example, is required for the SLG thin films. The bulk-NCG thin films are grown with a previously established process [37,38,39], in a CH${}_{4}$:H${}_{2}$ (60:75 sccm) atmosphere, at a pressure of 200 Pa and an RF discharge power of 100 W. The substrate temperature is kept at a high value of 890–900 °C, over a two h plasma growth step, to promote a high nucleation density. This in turn will result in thin films with high edge defect density, which is beneficial for sensors that rely on conductivity variations due to particle surface adsorption [37]. The PECVD process yields bulk-NCG thin films of ≊360–380 nm thickness with a conductivity of over $10^{4}$ S m${}^{-1}$ [38,39]. For the GNW thin films, the discharge power is increased to 300 W and the process pressure is lowered to 40 Pa to stimulate the growth-induced vertical morphology. The CH${}_{4}$ gas flow rate is also considerably lowered to 10 sccm and molecular hydrogen is removed from the precursor list. To stabilize the plasma and improve the hydrocarbon dissociation rate, Ar is introduced in a 190:10 (Ar:CH${}_{4}$) ratio [16]. The GNW thin films are grown at a 750 °C substrate temperature to obtain a specific surface area, suitable for functionalization. A more detailed description of the growth mechanism and the physical processes involved in the PECVD growth of GNW and bulk-NCG films can be found in refs. [15,16], and for SLG in refs. [34,35].

### 3.2. GFET Microfabrication

The GFETs’ fabrication process differs from the silicon-based FETs’ process as it entails a more complex series of photolithographic steps revolving around both wet and dry etching. The microfabrication of the graphenic-based FETs involves the deposition of the carbonic materials on a thermally oxidized Si wafer, either directly (i.e., GNW, bulk-NCG) or by transfer (i.e., SLG), followed by the metallic contacts’ deposition (i.e., back gate, source, drain, top gate) and functionalization of the source-drain channel.

#### 3.2.1. Technological Workflow

All presented GFETs were manufactured on $4^{\prime \prime }$ Si wafers with <100> crystallographic orientation that were thermally oxidized in an O${}_{2}$:H${}_{2}$ atmosphere at 900 °C for 340 min. The wafers were cleaned before and after the oxidation process by immersion in a Piranha solution (H${}_{2}$SO${}_{4}$:H${}_{2}$O${}_{2}$), washed in isopropyl alcohol, rinsed in deionized water, and thermally dried. Before undergoing the photolithographic processes, the wafers were dehydrated for 1 min at 150 °C in a hexamethyldisilazane (HMDS) atmosphere to promote enhanced adherence in the following steps.

**Figure 2:**
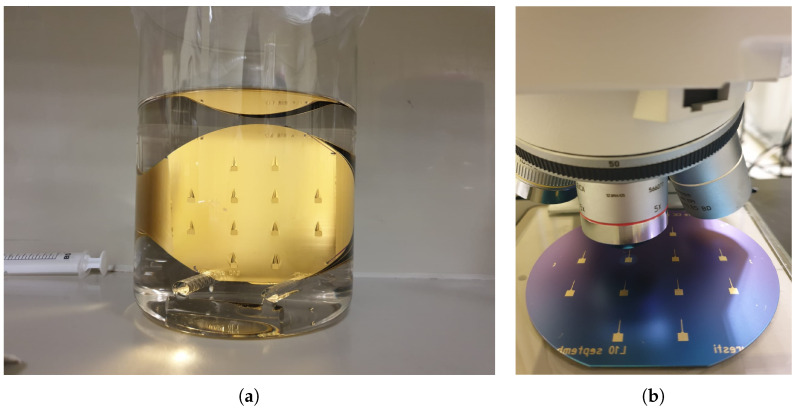
The back-side of a GFET wafer sample: (**a**) during and (**b**) after the back gate lift-off process.

In preparation for the back gate deposition, the back-side SiO${}_{2}$ layer is selectively etched down to a 50 nm thickness in the areas of interest by photolithographic masking with an HPR photoresist and wet chemical etching. During this step, the front side of the wafer is protected with a photoresist. All wafers are then cleaned in a Piranha solution and the GNW and bulk-NCG thin films are grown via PECVD as previously described, leaving the SLG film to be transferred at a later stage. The back gate contact consists of a 300 nm thick Au layer with an intermediate 30 nm Cr adhesion layer, both of which are deposited through e-beam evaporation with the Neva EVD-500A system (Neva, Japan). The contacts are deposited directly on LOR 5A and HPR 504 photoresists, which are already patterned through standard photolithographic processes, after which the wafers undergo the lift-off procedure in an ultrasonic acetone bath (Figure 2). The wafers are then further immersed in acetone for 18 h and cleaned with the AZ 400 K developer and isopropyl alcohol to ensure complete removal of the photoresists.

**Figure 3:**
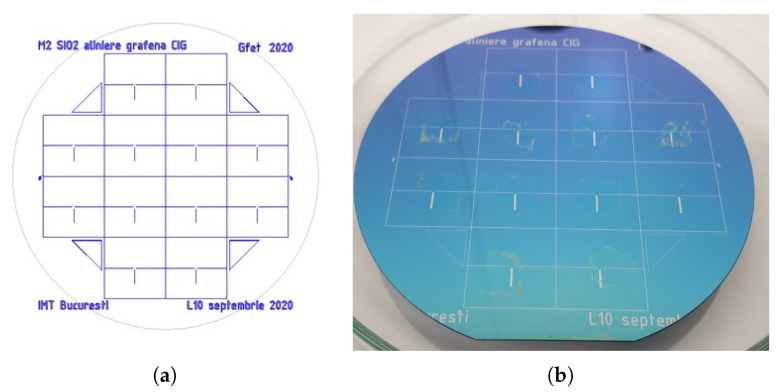
(**a**) Schematic of the mask used for patterning the alignment marks prior to single-layer graphene (SLG) transfer. (**b**) Wafer after successful transfer of the SLG thin films.

In the case of the SLG-based GFETs, an additional step is required to properly identify the areas onto which the SLG thin films are to be transferred. This is accomplished by wet chemical etching, using a specifically designed mask (Figure 3a) within a photolithographic process. The SLG thin films, initially grown on Cu foils, are then transferred through a series of wet chemical processes (Figure 3b) [34,35,36].

**Figure 4:**
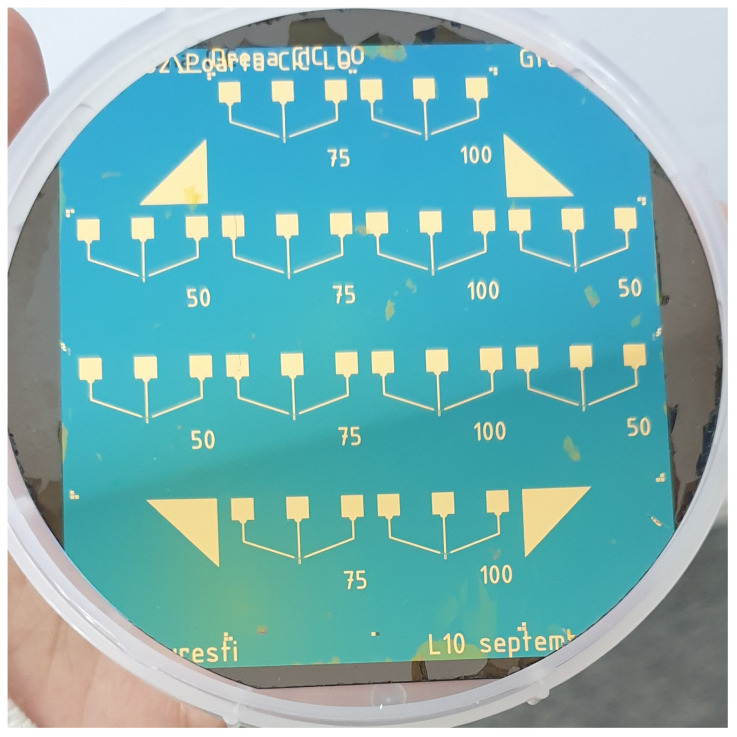
Photograph of bulk nanocrystalline graphite (bulk-NCG)-based GFETs on a $4^{\prime \prime }$ Si wafer after a complete run of the presented photolithographic technological workflow.

Next, the source and drain electrodes are deposited through e-beam evaporation and lift-off techniques similar to the back gate deposition, except this time a 365 nm thick Au layer and a 30 nm thick Cr adhesion layer are used. Following the subsequent cleaning in acetone and isopropyl alcohol, the graphenic source-drain channels on all wafers are masked with an AZ 4562 photoresist and the rest of the graphenic material is dry etched in an oxygen plasma. The channel patterning is carried out in a capacitively-coupled RF reactor with the Plasma Etcher—Etchlab 200 (Sentech Instruments, Germany) equipment, at 250 W RF discharge power, a pressure of 20 Pa, and an O${}_{2}$ flow rate of 50 sccm. After the photoresist is removed and the wafers are cleaned in acetone and isopropyl alcohol, a 30 nm thick passivation layer of Al${}_{2}$O${}_{3}$ is deposited and patterned through atomic layer deposition (ALD) and lift-off, respectively, where the top gate will be. Finally, a 335 nm thick Au layer with a 30 nm thick Cr adhesion layer are deposited and patterned through e-beam evaporation and lift-off, respectively, as the top gate electrode. A representative photograph of one of the wafers, after the complete series of photolithographic processes, is presented in Figure 4. After their successful processing, the samples are diced in individual GFETs for further functionalization and investigations.

#### 3.2.2. Source-Drain Channel Functionalization

The functionalization of the graphenic drain-source channel is accomplished by AuNPs decoration. The AuNPs’ purpose is to enhance the electron transfer rate and to provide a larger contact surface, thusly improving the sensor’s response.

**Figure 5:**
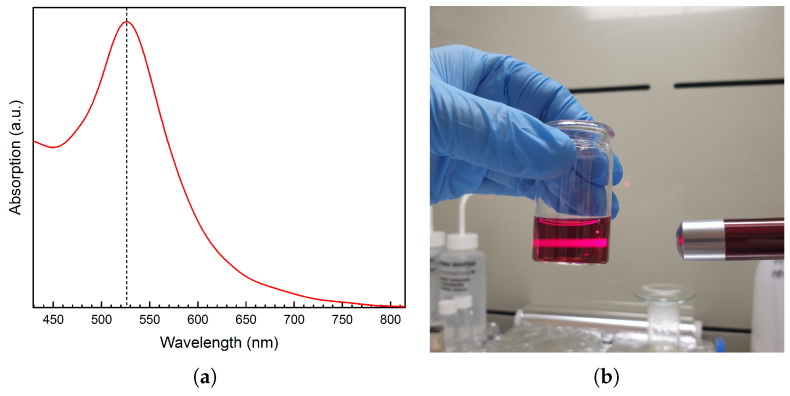
(**a**) UV-vis absorption spectra and (**b**) red laser light diffusion of the Au nanoparticles (AuNPs) colloidal solution. The dashed line in (**a**) represents the absorption peak situated at 526 nm.

The AuNPs were chemically synthesized by Au (III) to Au (0) reduction: 10 mL of chlorauric acid (HAuCl${}_{4}$) solution ($10^{-4}$ M/0.1 mM) (Sigma-Aldrich, Darmstadt, Germany) is heated up to boiling temperature and 200 μL of trisodium citrate (Na${}_{3}$C${}_{6}$H${}_{5}$O${}_{7}$) (Sigma-Aldrich, Germany) of 1% concentration is added under stirring at 300 rpm. The solution is maintained at a boiling temperature for five minutes, then cooled down to room temperature. The presence of AuNPs in the colloidal solution was investigated by UV-vis spectroscopy, with the Hitachi U-0080D UV–vis spectrometer. Absorption was measured in the 200–800 nm wavelength range and deionized water was used as a blank. Their successful synthesis is confirmed by the absorption peak present at a wavelength of 526 nm, as shown in Figure 5a. The AuNPs presence is also evidenced by light diffusion when directing a red laser through the solution (Figure 5b), the diffusion being possible due to the plasmonic resonance of the nanoparticles.

**Figure 6:**
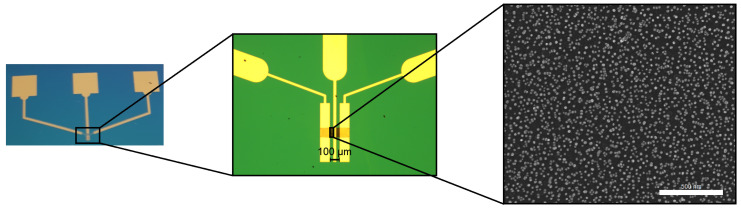
Final GFET structure (from **left** to **right**): a photograph of the top electrodes, an enhanced optical microscope image of the source-drain channel, and a scanning electron microscopy (SEM) micrograph at 130,000× magnification, showcasing the AuNP decoration of the channel.

For the graphenic channel’s decoration, the GFETs are immersed in the AuNPs colloidal solution overnight, then rinsed in deionized water and dried at 60 °C for two h. Scanning electron microscopy (SEM) investigations confirm the binding of 26.32±3.9 nm AuNPs at the surface of the carbonic channel (Figure 6).

## 4. GFET Operating Principle

The graphenic-based channel is deposited on a dielectric layer (i.e., SiO${}_{2}$) and the transducer is functionalized via AuNPs. The graphenic material is located between the source and drain electrodes, and the device is polarized by a back and front gate. The gate’s polarization creates the electric field that modulates the concentrations of charge carriers and the current between the source and drain electrodes ($I_{SD}$).

In silicon devices, the electrical current mostly flows either through electrons or holes, while the GFET allows conduction to take place equally through both electrons and holes, having an ambipolar behavior. This is evidenced by the presence of two conduction curves in the graphitic-built source-drain channel. One of them represents the electron flow through the channel when the top gate is positively biased and the other curve represents the hole flow when the top gate is negatively biased. The two conduction curves meet at the Dirac point or at the point of charge neutrality, which should theoretically be at zero voltage. However, in practice, the actual Dirac point may change depending on doping, the level of impurities on the graphenic surface, the ambient atmosphere, the biofunctionalization of the material, and other conditions. The influence on the channel’s free load by the potential applied to the gate can be translated to the gate control of the conductivity between the source and drain electrodes. Therefore, the GFET can have an n- or p-type channel depending on the potential applied on the gate, in contrast to classical FETs.

The use of a thin insulating material under the top gate improves the GFETs’ parameters, such as open circuit gain, transconductance ($g_{m}$), direct transmission coefficient, and biodetection sensitivity, expanding the GFETs’ area of applications as a result and allowing for very high operating frequencies. While the crystalline structure of traditional semiconductor materials has some limitations that cause them to dissipate more heat at higher frequencies, the hexagonal structure of graphene and graphene-based nanostructures, the high electron mobility, and several other factors, allow the GFETs to perform much better at very high frequencies. Thus, the transistors should theoretically have very high-speed switching capabilities, several times faster than silicon-based FETs.

The detection capabilities and mechanism of the GFETs can be investigated by applying a constant voltage between the source-drain electrodes ($V_{SD}$) and gate electrodes ($V_{G}$), and measuring the $I_{SD}$ currents over time. When there is no change in temperature, humidity, present chemicals, or other environmental factors, the measured current will be constant over time. If a solution (either a buffer or saline solution, such as blood, saliva, or blood serum) that contains electrically charged target biomolecules is attached to the channel after a set time (known as the detection time), some of the biomolecules will attach to the channel and the channel’s charge carrier concentration will change to compensate for the additional electrical charge. As a result, the conductivity of the device and the measured current will change according to the equation:

(1) σ≈enμ,

where σ is the electrical conductivity, *e* represents the charge of the electron, *n* is the electron concentration, and μ is the electron mobility. Equation (1) impresses on the variation of electron concentration at the interface between the channel’s surface and the electrically charged molecules, while also putting into perspective the linear dependence of the electrical conductivity on said concentration. Furthermore, the intensity of the electric current between source and drain will vary with the electrical conductivity, as per the following equation:

(2) $$I_{SD}=\frac{\sigma Wt}{L}V_{SD},$$

where *W*, *t*, and *L* are the width, thickness, and length of the channel, respectively, and $V_{SD}$ represents the electrical voltage between the source and the drain. By incorporating Equation (1) into Equation (2), the following relation is obtained for the current between the source and drain electrodes:

(3) $$I_{SD}\approx \frac{en\mu Wt}{L}V_{SD}.$$

The top gate potential electrostatically modulates the concentration of electric charge carriers in the graphenic channel. If a positive potential is applied to the top gate, the Fermi level shifts from the Dirac point into the conduction band, resulting in an n-type channel. If a potential is applied between the source and the drain in this case, the generated electric current in the channel is the result of an electron flow. The same effect occurs if a negative potential is applied at the top gate. This time the Fermi level drops into the valence band, resulting in a p-type channel with a hole-generated current when a potential is applied between the source and drain electrodes.

The source-drain current is defined as the ratio of channel charge, *Q*, to channel charge transit time, $T_{tr}$:

(4) $$\begin{array}{c} I_{SD}=-\frac{Q}{T_{tr}}, \end{array}$$

(5) $$\begin{array}{c} T_{tr}=\frac{L}{\nu_{d}}=\frac{L^{2}}{\mu V_{SD}}, \end{array}$$

(6) $$\begin{array}{c} Q=-C_{ox}\left(V_{G}-V_{DP}\right)WL, \end{array}$$

where $\nu_{d}$ is the drift speed, $C_{ox}$ is the top gate dielectric’s capacitance, and $V_{DP}$ represents the Dirac point potential (the neutrality point). For small drain potentials, the channel’s conduction is proportional to the top gate potential, and the source-drain current varies linearly with the source-drain potential, as described by:

(7) $$I_{SD}=-\frac{Q}{T_{tr}}=\mu \frac{W}{L}C_{ox}\left(V_{G}-V_{DP}\right)V_{SD}.$$

Moreover, for small drain potentials, as the drain current varies linearly with the drain potential and the channel’s conduction is proportional to the top gate voltage, the charge carrier mobility can be determined by:

(8) $$\mu =\frac{L}{W}\frac{1}{C_{ox}V_{SD}}\frac{dI_{SD}}{dV_{G}},$$

where $\frac{dI_{SD}}{dV_{G}}=g_{m}$ is the transconductance, or the slope of the $I_{SD}=f\left(V_{G}\right)$ characteristic.

## 5. Results and Discussion

**Figure 7:**
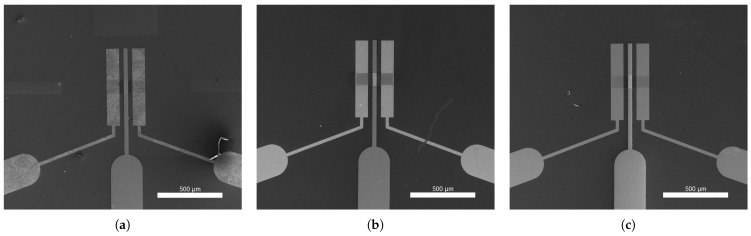
SEM micrographs at 130× magnification, displaying the top-view illustration of the GFETs’ graphenic channel: (**a**) SLG, (**b**) graphene/graphite nanowalls (GNW), and (**c**) bulk-NCG.

In order to confirm the successful realization of the GFETs’ modified channel and for their subsequent morphological characterization, SEM was employed via the Nova NanoSEM 630 (FEI Company, Hillsboro, OR, USA) at an acceleration voltage of 15 kV. Top-view SEM micrographs showcasing the graphenic source-drain channel and its respective electrodes, along with the top gate electrode, are presented in Figure 7 for all three types of GFETs (i.e., SLG, GNW, and bulk-NCG).

**Figure 8:**
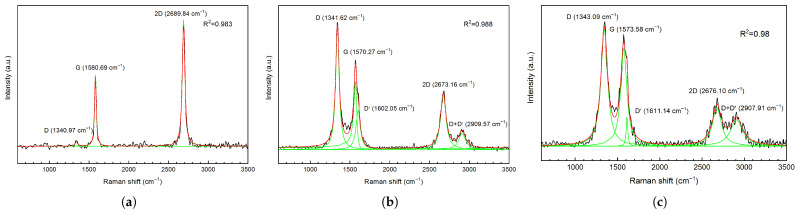
Raman spectra of the (**a**) SLG, (**b**) GNW, and (**c**) bulk-NCG graphenic channels of the fabricated GFETs. The black lines represent the processed experimental data, the red lines represent the cumulative fit, and the green lines represent the individual fitted peaks.

Additionally, Raman spectroscopy was used to evaluate the source-drain channel and analyze the structure of the different graphenic materials. The spectra (Figure 8) were acquired with the Raman Module Witec Alpha 300S (Witec, Ulm, Germany), equipped on a high-resolution Scanning Near-Field Optical Microscope at a 100× magnification and in conjunction with a 532 nm wavelength laser.

All Raman spectra are initially vector normalized, and a Savitzky–Golay filter is applied over an 11-point window with a third-order polynomial to improve the signal-to-noise ratio. The spectra are then background corrected by linear subtraction and deconvoluted into individual Lorentzian peaks, with the exception of the G peak in the case of the GNW and bulk-NCG films, which is described by a Breit–Wigner–Fano shape [40]. The coefficient of determination for the proposed cumulative fits is ≥98%. The SLG Raman spectrum shows the specific G band at ≊1580 cm${}^{-1}$, due to the sp${}^{2}$ hybridization of carbon, and a high intensity 2D band at ≊2690 cm${}^{-1}$, corresponding to the second order of zone-boundary phonons [41]. Typically, the 2D peak is four times more intense than the G peak in pristine graphene sheets [41,42]. The relatively smaller ratios of $A_{2D}/A_{G}≊2.66$ and $I_{2D}/I_{G}≊1.76$, where $A_{X}$ and $I_{X}$ are the integrated area and the intensity of peak *X*, respectively, could be explained by defects induced during the transfer process. The appearance of transfer-induced defects is also corroborated by the presence of a small intensity D peak, which can be observed at ≊1340 cm${}^{-1}$. The nanocrystalline structure of the GNW and bulk-NCG thin films can be ascertained from the sharp increase of the D band’s intensity, the sub-unitary value of the $I_{2D}/I_{G}$ ratio, a shift towards lower wavenumbers of the G peak, the appearance of bands D${}^{\prime }$ and D + D${}^{\prime }$, and a general broadening of all Raman bands [41,42,43]. Moreover, the steep increase of the 2D band’s full width at half maximum (FWHM), from ≊80 cm${}^{-1}$ for GNW to ≊127 cm${}^{-1}$ for bulk-NCG, suggests an increase in layer stacking and in the turbostratic relation between nanodomains [43]. In addition, the broader G band of bulk-NCG (FWHM${}_{G}$≊87 cm${}^{-1}$), compared to that of GNW (FWHM${}_{G}$≊45 cm${}^{-1}$), points towards a higher defect density.

**Figure 9:**
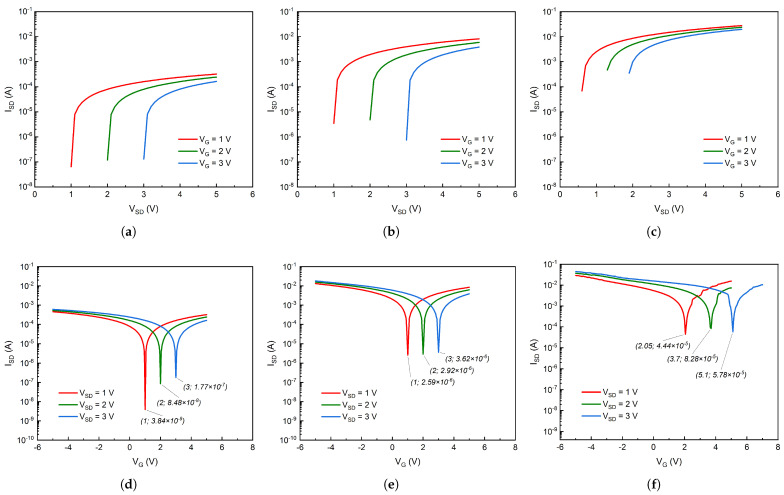
(**a**–**c**) Source-drain current variation with respect to the source-drain potential at different gate voltages and (**d**–**f**) *I*-*V* transfer curves at different source-drain voltages for the (**a**,**d**) SLG, (**b**,**e**) GNW, and (**c**,**f**) bulk-NCG based FETs.

The electrical performance of the GFETs was investigated at room temperature using the Keithley 4200-SCS Semiconductor Characterization System (Keithley Instruments, Cleveland, OH, USA) coupled with the Manual Wafer Probing Station EP6 (SüssMicroTec, Garching, Germany). Initially, several attempts were made with varying back gate voltages but, as depicted in Figure 1, the back gate electrode was ultimately grounded to avoid inserting additional variables (i.e., back gate oxide capacitance) and provide a more stable *I*-*V* readout. The output characteristics of the GFETs at different gate voltages, presented in Figure 9a–c, show that the source-drain current varies linearly with the source-drain potential for all investigated devices and reveal a significant increase in electrical conductance from the SLG to the GNW, and finally to the bulk-NCG-based FETs.

**Figure 10:**
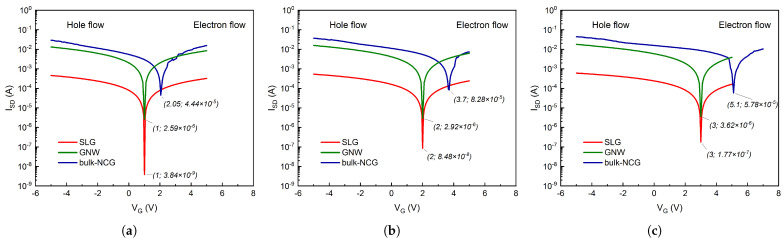
Relative transfer characteristics of all three GFETs for a source-drain voltage: (**a**) $V_{SD}=1$ V, (**b**) $V_{SD}=2$ V, and (**c**) $V_{SD}=3$ V.

The GFETs are unique in that they can act as both n- or p-type channel transistors, depending on the top gate applied voltage. The critical transition between the two regions of the GFETs’ *I*-*V* transfer curves can be observed in the case of all three materials (Figure 9d–f). At the Dirac point, the number of charge carriers is minimal, as is the current between the source and the drain, called the OFF current. The region where the current tends to saturate and $I_{SD}$ is almost independent of $V_{G}$ is called the ON current. The transfer characteristics show the relative electron and hole contributions to the source-drain current for all three different types of GFETs. At top gate voltages above the Dirac voltage ($V_{G}>V_{DP}$), the Fermi level is situated in the conduction band, and the total current is dominated by electron current. Below the Dirac voltage ($V_{G}<V_{DP}$), the Fermi level is located in the valence band, and the total current is dominated by hole current. Around $V_{G}≊V_{DP}$, the total current is a combination of electron and hole currents with equal contributions. All fabricated GFETs display an ambipolar behavior modulated by the gate voltage, as displayed in Figure 10. In the case of the bulk-NCG-based GFETs, the Dirac point appears to be shifted towards higher values in all three cases (i.e., $V_{SD}=1,\;2,\;\;$and 3 V). This positive shift can be mainly attributed to the higher defect density of the bulk-NCG thin film, as was shown from the Raman analysis.

Another important distinction between the three graphenic channels that can be ascertained from Figure 10 is their minimum current intensities at their respective Dirac points, i.e., the OFF current, with $I_{OFF}$ (SLG) <$I_{OFF}$ (GNW) <$I_{OFF}$ (bulk-NCG). The SLG-based channel displays the lowest minimum current intensity despite having the lowest defect density out of the three types of graphenic films. Defects induced during the transfer phase of the SLG film may be a contributing factor, but this is primarily due to the film’s atomically thin nature, which makes it more susceptible to the substrate’s influence [26], as mentioned in Section 2. The former statement is additionally reinforced by the increasing values of the SLG channel’s $I_{OFF}$ as the source-drain voltage is increased. The OFF current for the SLG channel increases with one order of magnitude, from $10^{-9}$ to $10^{-8}$, and ultimately to $10^{-7}$ A as $V_{SD}$ is increased from 1 to 2, and finally to 3 V, respectively, while it remains relatively constant at an order of $10^{-6}$ and $10^{-5}$ A for the GNW and bulk-NCG channels, respectively. Concerning the other two graphenic FETs, the bulk-NCG GFET displayed higher ON and OFF currents than the GNW-based FET, despite having a higher defect density, as was showcased by the Raman analysis. We believe this is directly related to their intimate morphology and the nanocrystallites’ stacking and orientation. As stated in the Section 1, the bulk-NCG’s nanodomains are more densely packed and the films have smoother surface finish, which allows for better conduction on a direction parallel to the SiO${}_{2}$ underlayer, while the nanocrystalline domains of the GNW films gravitate towards a perpendicular direction with respect to the substrate (i.e., c-axis parallel to the SiO${}_{2}$ underlayer). The perpendicular configuration of the graphenic planes, which are in turn somewhat parallel to each other, may hinder charge conduction in the horizontal direction due to gaps between individual walls and drive the charge carriers to the lower basal planes where they might find better conduction pathways but be more susceptible to substrate interference, as in the case of the SLG films. Additionally, their very high specific surface area with high edge density may lead to an increased current leakage. All these factors coupled together lead to the bulk-NCG channel exhibiting a better electrical conduction in the present electrode configuration.

The measured transconductance values of the GFETs and the charge carrier mobility, calculated using Equation (8), are showcased in Table 2. The transconductance of the bulk-NCG source-drain channel is more than 60 times higher than that of the SLG source-drain channel and double that of the GNW channel. In addition, the mobility of electric charge carriers for the bulk-NCG source-drain channel is more than 100 times higher than that of the SLG source-drain channel and more than 2 times higher than that of the GNW channel.

**Figure 11:**
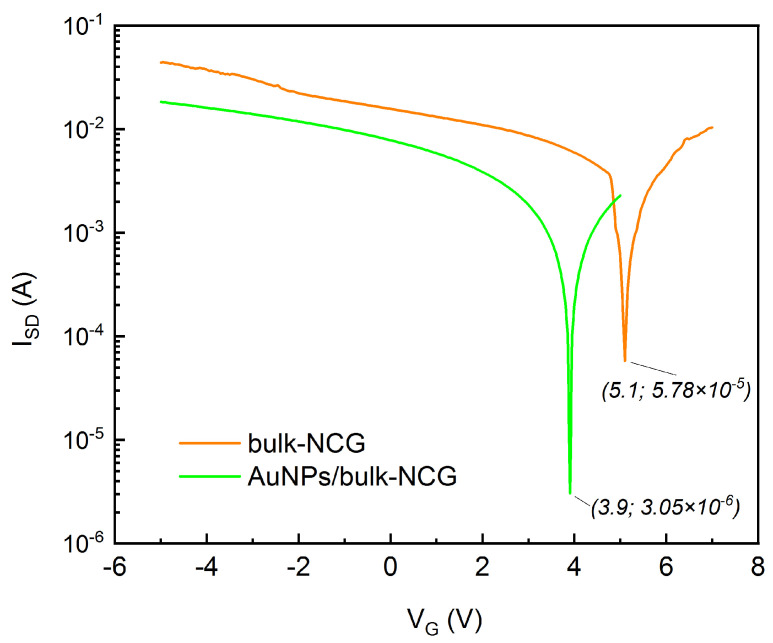
*I*-*V* characteristics measured at a source-drain potential of $V_{SD}=3$ V for a bulk-NCG FET before and after AuNP decoration.

As the bulk-NCG channels showed the best electrical characteristics, further investigations were employed in an attempt to improve their sensitivity by AuNP decoration. When comparing the *I*-*V* transfer characteristics of the source-drain channel before and after AuNPs decoration, we notice an improved sensitivity. The Dirac point of the bulk-NCG FET showed a −1.2 V shift as the AuNPs surface is negatively charged, and an increase of the ON/OFF current ratio ($I_{ON}⁄I_{OFF}$) from ≊178.95 to ≊746.43 (Figure 11).

## 6. Conclusions

Graphenic materials have proven to be valuable assets in the fabrication of FETs for sensing applications. In addition to their close relation to atomically thin graphene, in terms of their composition and molecular structure, which relate to similar physico-chemical properties, they provide a more feasible fabrication process for the respective GFETs. The PECVD synthesis of the graphenic materials is accomplished directly on an insulating layer, therefore eliminating the requirement of a transfer step from the technological workflow, a step that is still necessary for SLG. Their shared sp${}^{2}$ hybridization, as well as their individual forms of crystallinity, are derived from their Raman spectra. Both of these factors play a major role in their respective conduction mechanisms. All of the investigated devices show both n- and p- type characteristics depending on the applied gate voltage, and the comparative transfer curves show a considerable shift of the Dirac point for the source-drain channel made of bulk-NCG in comparison to the SLG and GNW channels. Additionally, the decoration of the source-drain channel with AuNPs significantly improves the GFETs’ sensitivity. The Dirac point behavior of the bulk-NCG channel would suggest the possible existence of a band gap in the material and could allow for additional sensitivity improvement in the future. This comparative study illustrates the electrical behavior of three graphenic materials with a similar intra-molecular structure and showcases how their different inter-molecular structure can affect their electrical performance. Despite having an increased defect density, the bulk-NCG channel’s internal stacking allows it to outperform the other two graphenic channels in terms of electrical conductivity, in the present electrode architecture. Although the GNW channel displays a poorer electrical conductivity than the bulk-NCG one, its higher specific surface area may prove advantageous for biosensing if a more favorable electrode configuration is found. Subsequent studies will entail a more comprehensive investigation of nanocrystalline graphite’s conduction mechanism and of the GFETs’ response after both AuNP functionalization and anti-body conjugation of the source-drain channel.

## Figures and Tables

**Table 1:** Graphenic materials’ growth process parameters.

Thin Film	Temperature (°C)	Time (min)	RF Power (W)	Pressure (Pa)	Precursors
SLG	1080	60	−	400	Ar:H_2_:CH_4_
GNW	750	60	300	40	Ar:CH_4_
bulk-NCG	890	120	100	200	H_2_:CH_4_

**Table 2:** Transconductance and carrier mobility as a function of source-drain voltage for the three GFET types.

	$V_{\mathrm{SD}}=1$ V		$V_{\mathrm{SD}}=2$ V		$V_{\mathrm{SD}}=3$ V	
	$g_{m}$ (A V${}^{-1}$)	μ (cm${}^{2}$ V${}^{-1}$ s${}^{-1}$)	$g_{m}$ (A V${}^{-1}$)	μ (cm${}^{2}$ V${}^{-1}$ s${}^{-1}$)	$g_{m}$ (A V${}^{-1}$)	μ (cm${}^{2}$ V${}^{-1}$ s${}^{-1}$)
SLG	$8.3\times 10^{-5}$	$8.85\times 10^{1}$	$8.2\times 10^{-5}$	$1.745\times 10^{2}$	$8.2\times 10^{-5}$	$2.613\times 10^{2}$
GNW	$2.6\times 10^{-3}$	$4.14\times 10^{3}$	$2.2\times 10^{-3}$	$8.43\times 10^{3}$	$2.2\times 10^{-3}$	$1.263\times 10^{4}$
bulk-NCG	$4.6\times 10^{-3}$	$8.84\times 10^{3}$	$4.5\times 10^{-3}$	$1.713\times 10^{4}$	$4.9\times 10^{-3}$	$2.866\times 10^{4}$

## Data Availability

The data presented in this study are available on request from the corresponding author.

## Abbreviations

The following abbreviations are used in this manuscript

ALD	atomic layer deposition
CNW	carbon nanowalls
CVD	chemical vapor deposition
FET	field-effect transistor
FWHM	full width at half maximum
GDM	graphene-derived material
GFET	graphenic-based field-effect transistor
GNW	graphene/graphhite nanowalls
HMDS	hexamethyldisilazane
MOSFET	metal-oxide-semiconductor
NCG	nanocrystalline graphite
NPs	nanoparticles
PECVD	plasma-enhanced chemical vapor deposition
SEM	scanning electron microscopy
SLG	single-layer graphene
VG	vertical graphene
